# Transforming Growth Factor TGFβ Increases Levels of Microtubule-Associated Protein MAP1S and Autophagy Flux in Pancreatic Ductal Adenocarcinomas

**DOI:** 10.1371/journal.pone.0143150

**Published:** 2015-11-16

**Authors:** Kun Song, Wei Hu, Fei Yue, Jing Zou, Wenjiao Li, Qi Chen, Qizhi Yao, Weijia Sun, Leyuan Liu

**Affiliations:** 1 Department of General Surgery, Xiangya Hospital, Central South University, Xiangya Road 87, 410008, Changsha, Hunan Province, China; 2 Center for Translational Cancer Research, Institute of Biosciences and Technology, Texas A&M Health Science Center, Houston, Texas, United States of America; 3 The First People’s Hospital, Lian Yungang City, Jiangsu Province, China; 4 Department of Surgery, Molecular Surgeon Research Center, Baylor College of Medicine, One Baylor Plaza, Houston, Texas, United States of America; 5 Department of Molecular and Cellular Medicine, College of Medicine, Texas A&M Health Science Center, College Station, Texas, United States of America; Georgia Regents University, UNITED STATES

## Abstract

**Background and Aim:**

Autophagy is a cellular process to regulate the turnover of misfolded/aggregated proteins or dysfunctional organelles such as damaged mitochondria. Microtubule-associated protein MAP1S (originally named C19ORF5) is a widely-distributed homologue of neuronal-specific MAP1A and MAP1B with which autophagy marker light chain 3 (LC3) was originally co-purified. MAP1S bridges autophagic components with microtubules and mitochondria through LC3 and positively regulates autophagy flux from autophagosomal biogenesis to degradation. The MAP1S-mediated autophagy suppresses tumorigenesis as suggested in a mouse liver cancer model and in prostate cancer patients. The TGFβ signaling pathway plays a central role in pancreatic tumorigenesis, and high levels of TGFβ suggest a tumor suppressive function and predict a better survival for some patients with resectable pancreatic ductal adenocarcinoma. In this study, we try to understand the relationship between TGFβ and MAP1S-mediated autophagy in pancreatic ductal adenocarcinoma.

**Methods:**

We collected the tumor and its adjacent normal tissues from 33 randomly selected patients of pancreatic ductal adenocarcinomas to test the association between TGFβ and autophagy markers MAP1S and LC3. Then we tested the cause and effect relation between TGFβ and autophagy markers in cultured pancreatic cancer cell lines.

**Results:**

Here we show that levels of TGFβ and autophagy markers MAP1S and LC3 are dramatically elevated in tumor tissues from patients with pancreatic ductal adenocarcinomas. TGFβ increases levels of MAP1S protein and enhances autophagy flux.

**Conclusion:**

TGFβ may suppress the development of pancreatic ductal adenocarcinomas by enhancing MAP1S-mediated autophagy.

## Introduction

Autophagy-lysosome system is the major pathway to degrade damaged organelles, misfolded/aggregated proteins and other macromolecules in mammalian cells [1, 2]. Although autophagy is frequently quoted with apoptotic cell death, a balanced autophagy is essentially a cellular process to promote survival rather than cause death, only over-activated autophagy results in depletion of organelles and cell death or defective autophagy triggers accumulation of dysfunctional mitochondria and eventually robust oxidative stress [3]. Oxidative stress induces death of post-mitotic cells, but it is diluted through cell division in proliferated cells. Consequently, oxidative stress induces genome instability that is amplified through a series of autocatalytic karyotypic evolution through continuous cycles of cell division and chromosomal breakage-fusion-bridge and finally leads to tumorigenesis [4–6]. In general, autophagy suppresses tumorigenesis [7, 8].

Microtubule-associated protein MAP1S was originally named as C19ORF5 (chromosome 19 open reading frame 5). It was initially found to interact with RASSF1A, a microtubule stabilizer and tumor suppressor, and LRPPRC, a mitochondrion-associated autophagy inhibitor [9–13]. Similar to its homologue MAP1A and MAP1B, MAP1S interacts with LC3, a mammalian homologue of yeast autophagy marker ATG8 [14–18]. We identified MAP1S as a key regulator to positively enhance autophagy flux [18]. Based on a huge data set from The Cancer Genome Atlas, somatic mutations in MAP1S were found to be significantly associated with poor prognosis of patients suffering from ovarian cancer [19]. In a mouse model of chemical carcinogen-induced hepatocellular carcinomas, we found that the autophagy-defective MAP1S-deficient mice exhibit higher levels of genome instability and develop more tumor foci and higher malignance of hepatocellular carcinomas than the wild-type mice [20]. We concluded that an increase in MAP1S levels leads to an activation of autophagy to suppress genome instability so that both the incidence of hepatocarcinogenesis and malignant progression are suppressed. In addition, the protein levels of MAP1S and LRPPRC are closely associated with survival of patients with prostate adenocarcinomas [19, 21, 22]. Thus, a link between MAP1S-enhanced autophagy and suppression of genomic instability and tumorigenesis has been revealed.

Pancreatic ductal adenocarcinoma exhibits high levels of genome instability and is considered as one of the most aggressive human cancers [8]. The TGFβ signaling pathway emerges as a main regulator of pancreatic tumorigenesis [23]. Although TGF-β was reported to promote the progression of advanced tumors [24], it is widely accept as a potent growth inhibitor with tumor suppressive activity [25, 26]. Most of patients with pancreatic ductal adenocarcinoma have a very poor prognosis, but some patients with resectable pancreatic ductal adenocarcinoma have high levels of TGFβ and survive for long time [27]. TGF-β was reported to induce autophagy and promote the degradation of long-lived proteins to suppress hepatocellular carcinoma in humans [28]. Although TGFβ significantly increases the mRNA levels of autophagy regulatory genes such as Beclin 1, ATG5 and ATG7, it has no obvious impact on the protein levels of those regulators [28]. Thus, the mechanism by which TGFβ enhances autophagy flux has not been deciphered.

In the present study, we showed that levels of TGFβ, MAP1S and LC3 proteins were dramatically elevated in pancreatic cancer tissues, and TGFβ enhances autophagy flux through MAP1S to suppress the development of pancreatic ductal adenocarcinomas.

## Materials and Methods

### Collection of human tissue samples

Four male patients with pancreatic ductal adenocarcinomas were randomly selected from those enrolled in Xiangya Hospital, Central South University, Changsha, Hunan Province, China, during 2013. Each of them was treated by surgery and donated the pancreatic ductal adenocarcinoma tissues and respective adjacent normal ductal epithelium tissues from surgery. The adjacent normal tissues were usually collected at sites 2 cm away from the tumor tissues. Four sets of primary samples were taken from the patients, a portion of each sample was fixed in 10% formalin, embedded in paraffin, sectioned consecutively at 5 μm, stained with antibody and counterstained with hematoxylin and eosin similarly as previously reported [29]. The left portion from each sample was frozen in liquid nitrogen and homogenized to prepare lysates for immunoblot. To further confirm the results from four patients, we purchased the HPan-Ade060CS-01 tissue arrays from Shanghai Outdo Biotech Co., LTD which carry the pancreatic ductal adenocarcinoma tissues and respective adjacent normal grand and ductal tissues from 29 patients. The use of the clinical samples for analysis was approved by the Ethics Committee of the Central South University. The subjects gave full written informed consent, and patient anonymity has been preserved. This institutional review board specifically approved this study.

### Immunohistochemistry analysis

The sections were deparaffinized and rehydrated, and endogenous peroxidase was inhibited with 0.3% H_2_O_2_ in methanol. Slides were boiled in 0.01 M, pH 6.0 sodium citrate buffer for 15 min in a microwave oven to retrieve antigen. The slides were blocked with the 5% normal goat serum, stained with primary antibodies against TGFβ (ab66043 from abcam), MAP1S (4G1, AG10006, Precision Antibody), or LC3 (NB 100–2331, Novus Biologicals) in blocking buffer (1:100), and incubated at 4°C overnight. After incubation with biotinylated secondary antisera, the streptavidin–biotin complex/horseradish peroxidase was applied. Then, signals were visualized with 3,30-diaminobenzidine tetrahydrochloride and counterstained in hematoxylin as described [30]. Immunostaining results from the purchased tissue arrays and four samples collected in our hospital (total 33 patients) were scored by two independent pathological technicians following the standards as described [31]. Briefly, ten fields from each slide were subjected to counting the percentage of positive cells: 0, <5%; 1, 5–30%; 2, 31–60%; and 3, >60%. Immunostaining intensities were scored as 0 if no staining, 1 if color was pale yellow; 2 if color was yellow; and 3 if color was brown in positive cells. The scores of positive cells and scores of staining intensities were multiplied to generate new scores for each patients. The final scores were designated as “-” if the new score was 0, “+” if 1, “++” if new score was 2–4, and “+++” if new score was >4. The differences of immunostaining intensities between tumor tissues and their adjacent normal tissues, between different age groups, between male and female, or between different clinical stages were analyzed with the Wilcoxon signed-rank test.

### Cell transfection

Cell lines used for transfection included HeLa, HeLa cells stably expressing EGFP–LC3 (HeLa-GFP-LC3), PANC-1, Capan-2, wild-type or MAP1S^-/-^ MEF cells (mouse embryonic fibroblast) that were established as previously described [18, 32, 33]. Lipofectamine 2000 was used to pack plasmids and Oligofectamine was used to pack siRNA molecules following the manufacturer’s recommended protocols. Cells grown in twelve-well plates, six-well plates or 100-mm-diameter Petri dishes with or without coverslips were transfected with plasmids for 48 hrs. Similarly, cells grown to 30% confluence in twelve-well plates, six-well culture plates with or without coverslips were transfected with random sequences or MAP1S-specific siRNAs for 72 hrs. Cells on coverslips were fixed for direct observation of GFP-LC3 punctate foci under a Zeiss LSM 510 confocal microscope, while cells were harvested to prepare cell lysates for immunoblot analysis (six-well plates).

### Immunoblot analyses

Frozen tissues or collected cultured cell pellets were homogenized and proteins were isolated in RIPA buffer (150 mmol/L NaCl, 1.0% Triton X-100, 0.5% sodium deoxycholate, 0.1% SDS, and 50 mmol/L Tris–HCl; pH 8.0). The concentrations of total proteins in pancreatic tissue lysates were determined as described in detail [18]. After boiled lysates were centrifuged, the clear supernants were loaded on either 10 or 15% polyacrylamide gels containing SDS and acrylamide, and the gel concentrations were selected according to the molecular weights of the analyzed proteins. Proteins were resolved through electrophoresis and transferred to PVDF membranes. The membranes were then incubated with primary antibody against MAP1S, LC3 or β-Actin (SC-47778, Santa Cruz Biotechnology, Inc.), and protein bands were detected with enhanced chemiluminescence (ECL) based on horseradish peroxidase (HRP)-conjugated secondary antibodies (mouse #172–1011 and rabbit #172–1019, Bio-Rad) with the ECL Western Blotting Detection Reagents. ECL Western Blotting Detection Reagents and PVDF transfer membrane were purchased from GE Health. After exposure, x-ray films were developed, washed, dried and scanned into image files. The relative intensity of a protein band in a sample to its β-actin control was measured using ImageJ software (NIH).

### Detection of MAP1S mRNA levels by quantitative real-time polymerase chain reaction

Total RNA was extracted from PANC-1 or Capan-2 cells with the Trizol reagent (Invitrogene#15596–026) according to the manufacturer’s instructions. Reverse transcription using random primers was carried out with the Invitrogene SuperScript III First-Strand System. Real-time PCR was performed using the SYBR Premix ExTaq (TaKaRa RR820A). Primers for human MAP1S included a Forward Primer 5’-CGCTGGAAGAACTCCTCATC-3’ and a Reverse Primer 5’-GAGTGAGCCCAGTGAGAAGG-3’, and those for human β-Actin included a Forward Primer 5’-ACTCTTCCAGCCTTCCTTCC-3’ and a Reverse Primer 5’-CAGTGATCTCCTTCTGCATCC-3’. The Relative levels of MAP1S mRNA were quantified by a normalization of the amount of MAP1S to the amount of β-actin.

## Results

### Intensities of TGFβ, MAP1S and autophagy marker are uniformly elevated in human pancreatic ductal adenocarcinomas

**Fig 1:**
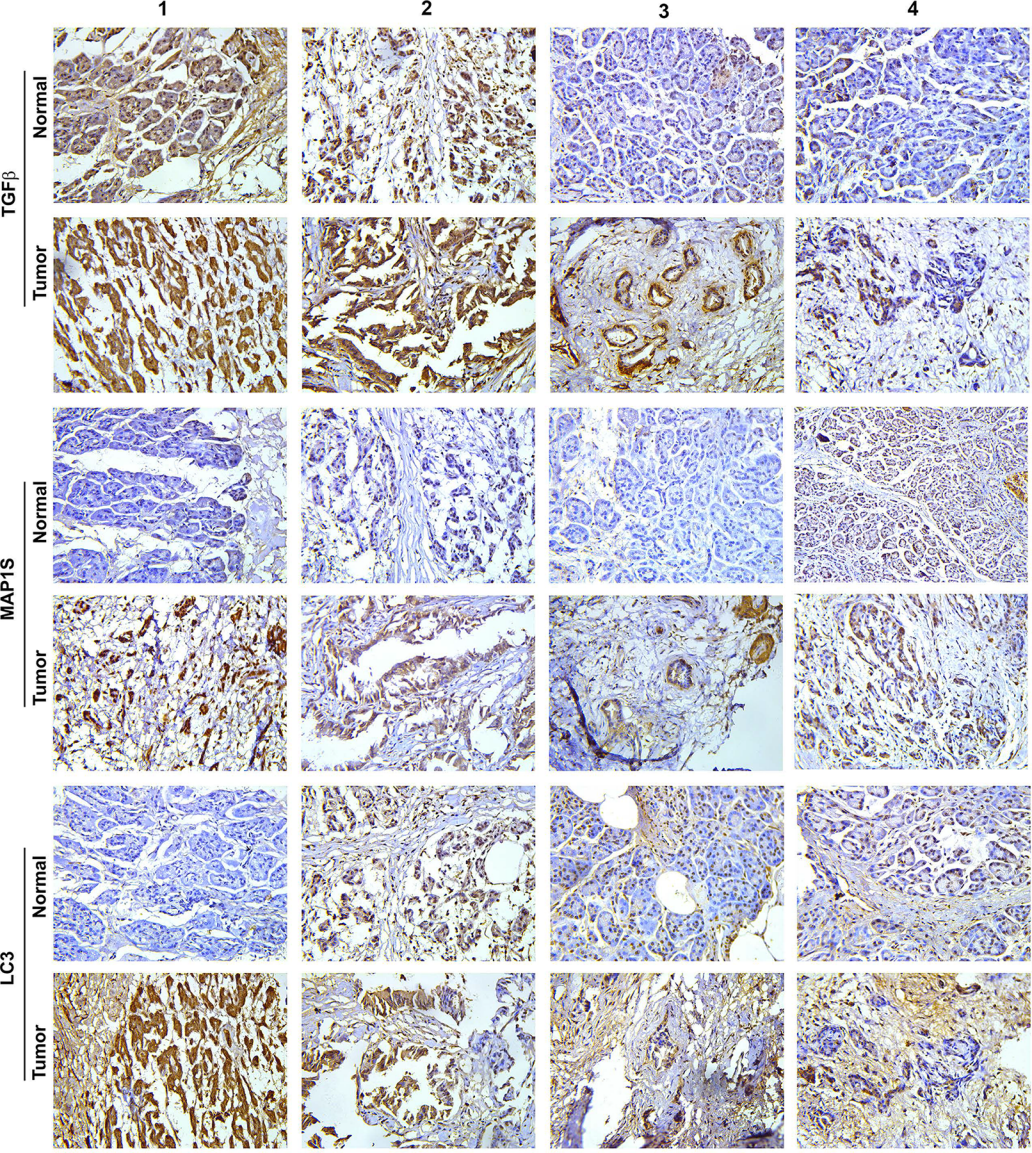
Intensities of TGFβ, MAP1S and autophagy are uniformly elevated in human pancreatic cancer tissues as detected by immunostaining analyses. Pancreatic cancer tissues and their adjacent normal tissues were collected from four patients who enrolled for pancreatic cancer treatment. The tissue sections were subjected to immunostaining with antibody against TGFβ, MAP1S or LC3, respectively.

**Fig 2:**
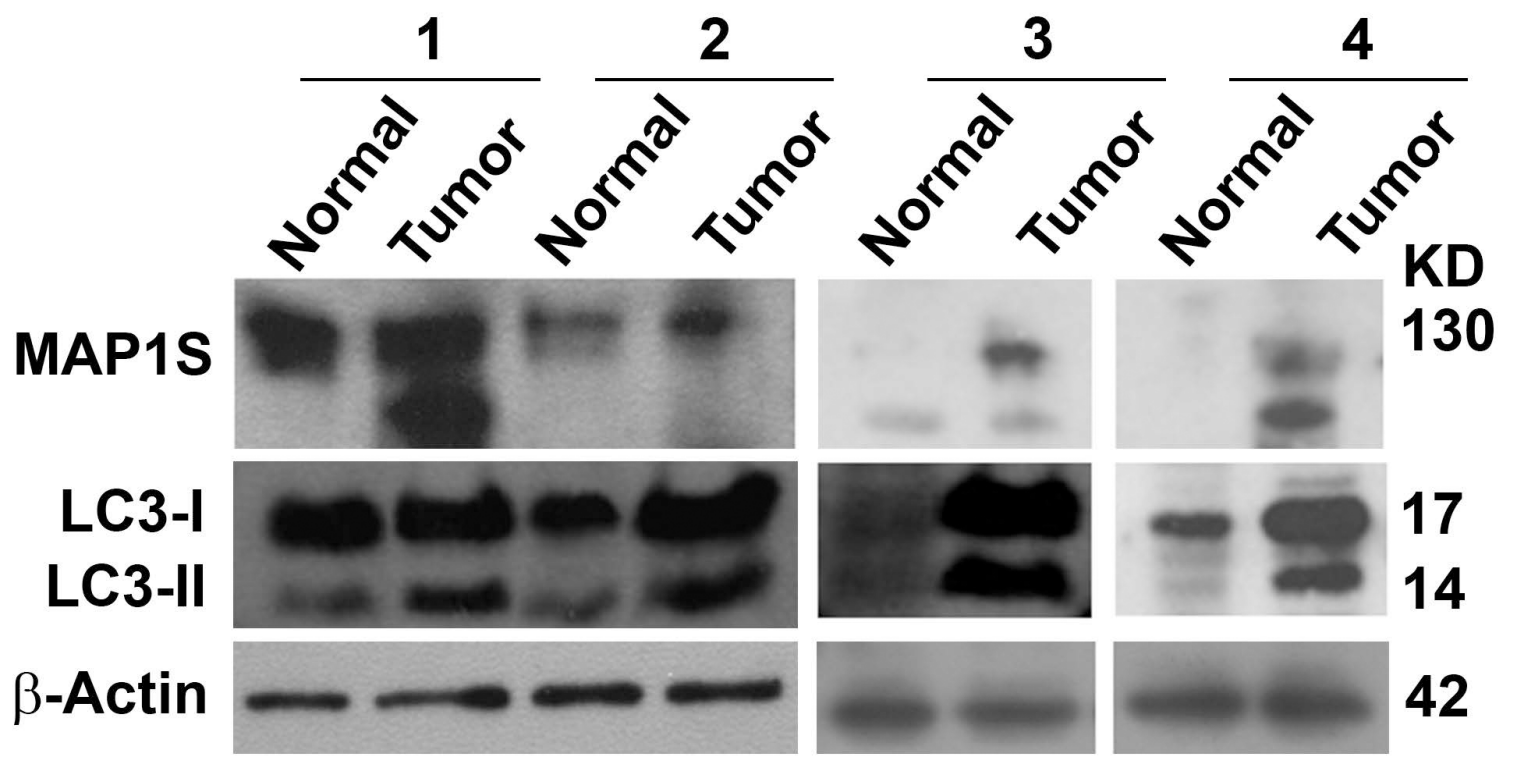
Intensities of MAP1S and autophagy flux are elevated in human pancreatic cancer tissues as detected by immunoblot analyses. The same pancreatic cancer tissues and their adjacent normal tissues as used in Fig 1 were analyzed. The tissue lysates containing the same amount of total proteins were subjected to immunoblotting with antibody against MAP1S or LC3, respectively. β-Actin served as another loading control.

**Fig 3:**
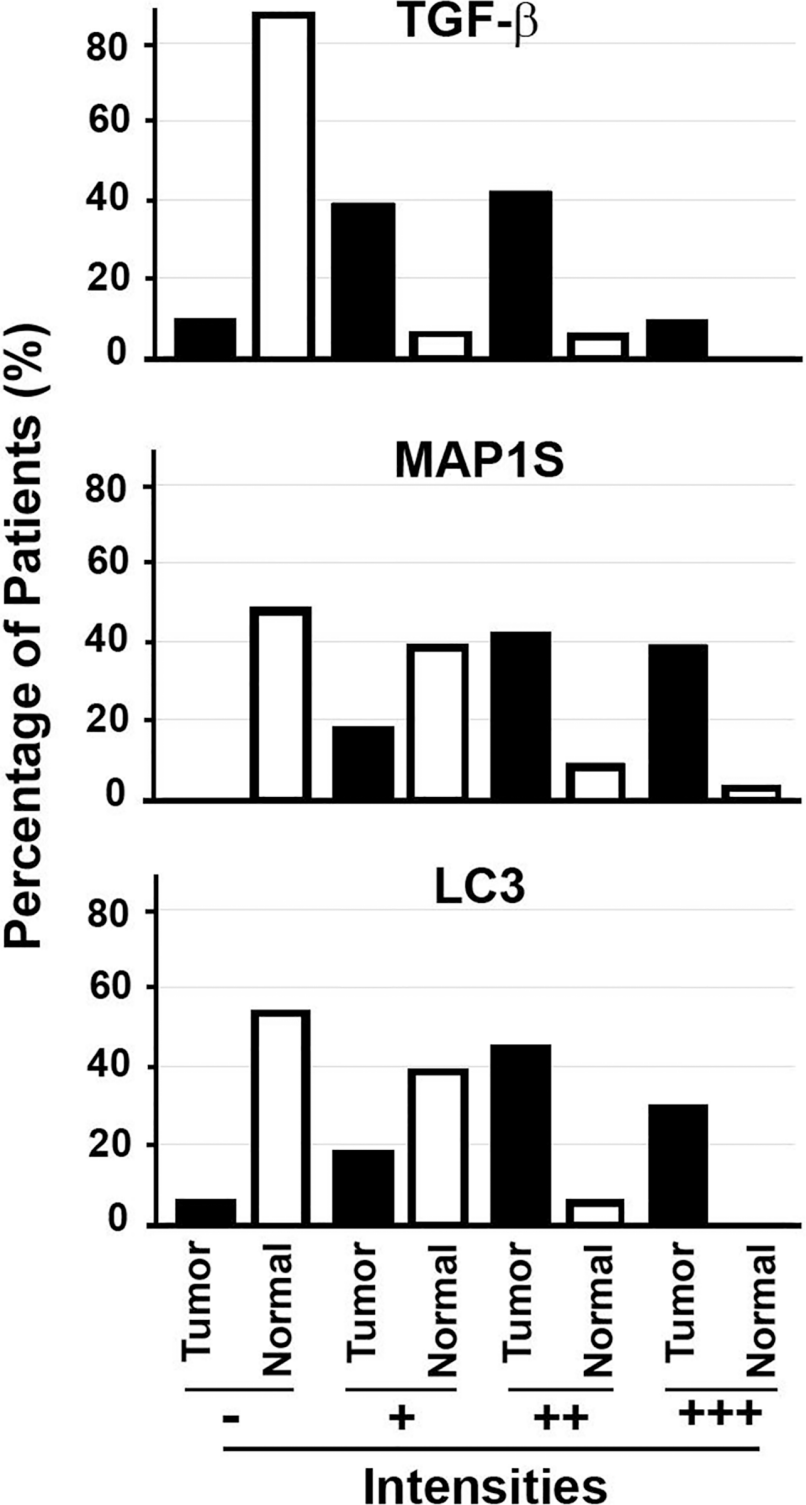
Intensities of TGFβ, MAP1S and LC3 are uniformly elevated in human pancreatic cancer tissues as detected by immunostaining analyses. Tissue arrays containing pancreatic ductal adenocarcinoma tissues and respective adjacent normal grand and ductal tissues from 29 patients were analyzed for their expression of TGFβ, MAP1S and LC3. The distribution patterns of different expression levels were compared between tumor and normal tissues of the combined population of 33 patients. The differences were significant with p≤0.001.

We collected fresh pancreatic tissue samples from four randomly elected patients diagnosed with pancreatic ductal adenocarcinomas from those who enrolled in Xiangya Hospital, Central South University, Hunan Province, China. The tumor tissues were isolated, and the nearby morphologically normal tissues were collected as patient-matched controls. Four sets of samples were subjected to immunostaining analysis. In general, the intensities of TGFβ, MAP1S and LC3 in tumors were higher than in the nearby normal tissues (Fig 1). Some of normal tissues exhibited significant staining of the related markers. The elevation in the intensities of MAP1S and autophagy markers in human pancreatic cancer was confirmed by immunoblot analysis of the same sets of normal and tumor tissue samples (Fig 2). Because of small sample size, we expanded our tests into tissue arrays made from 29 randomly selected patients. From the total 33 patients, the immunostaining intensities for all three proteins were uniformly higher in tumor tissues than in their adjacent normal tissues (Fig 3). Thus, the levels of TGFβ, MAP1S and autophagy marker LC3-II are coordinately increased during tumorigenesis.

### TGFβ enhances levels of MAP1S protein and autophagy flux in different pancreatic cell lines

**Fig 4:**
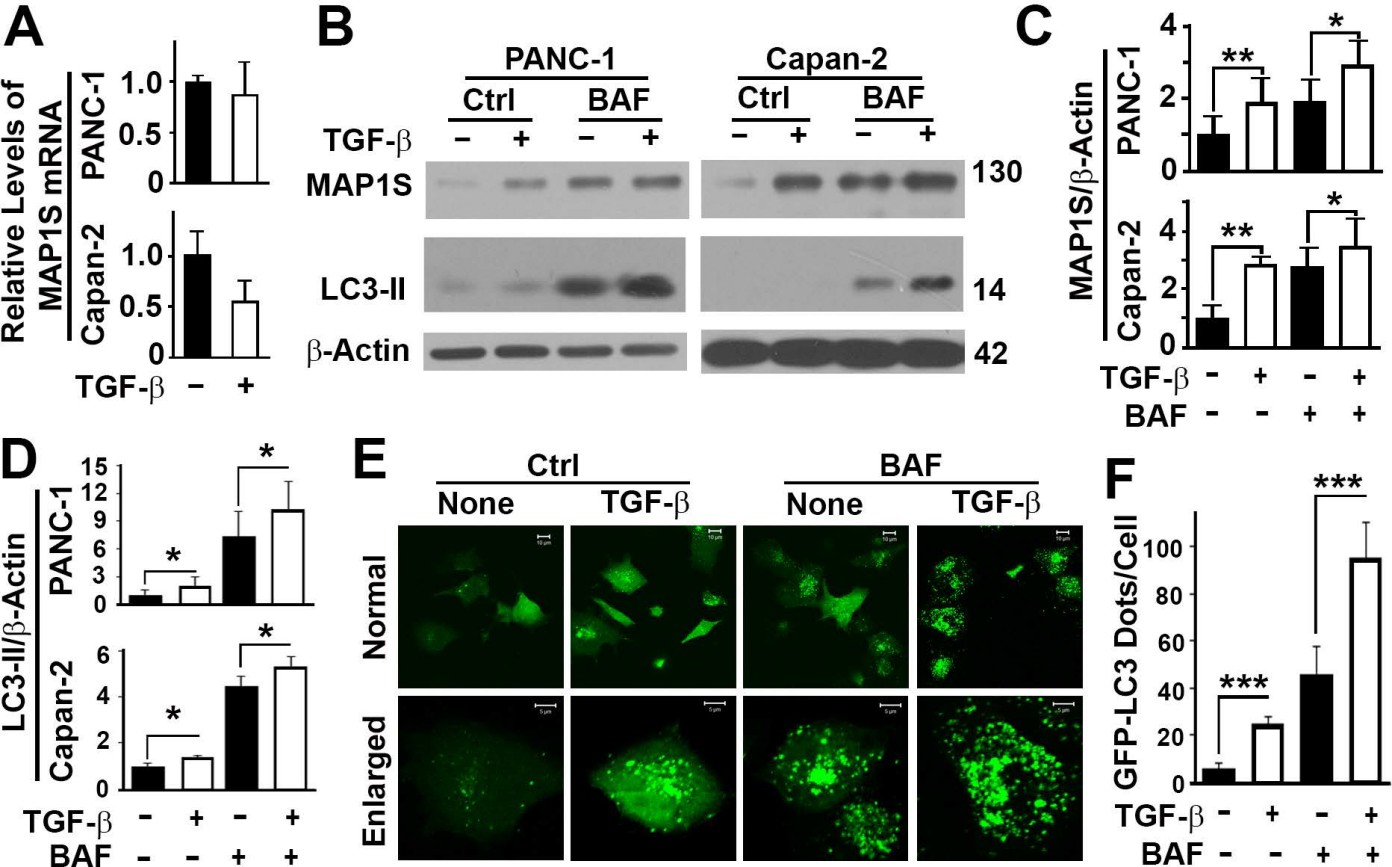
TGFβ enhances levels of MAP1S protein and autophagy flux in different pancreatic cell lines. A) A real-time RT-PCR analysis of the impact of TGFβ on the expression of *MAP1S* gene in pancreatic cancer cell line PANC-1 and Capan-2. The mRNA levels in untreated samples were set as 1. B) An immunoblotting analysis of the protein levels of MAP1S and LC3 in untreated or TGFβ-treated PANC-1 and Capan-2 cells in the absence (Ctrl) or presence of lysosomal inhibitor Bafilomycin A1 (BAF). Lysates with the same amount of total proteins were loaded in each lane and β-Actin served as another loading control. C,D) Plots of relative levels of MAP1S (C) or LC3-II (D) as shown as a representative in panel (B). The levels of MAP1S in untreated cells were set as 1. Results are the means ± S.D. of at least three repeats and the differences were compared using Student’s t test. **, P≤0.01; and *, P≤0.05. E) A fluorescent imaging analysis of the impact of TGFβ on PANC-1 cells transiently transfected with a plasmid for 48 hrs to express GFP–LC3 in the absence (Ctrl) or presence of TGFβ and/or BAF for 12 hrs. F) A quantification of GFP–LC3-labelled autophagosomes as shown in panel (E). The data were the average number of GFP-LC3 punctate foci ± S.D. for ten randomly selected images with size of 512 pixels×512 pixels for each that covers about 10 cells on average. The significance of differences was determined by Student’s t test. ***P≤0.001.

To understand whether the elevation in TGFβ levels and elevation in levels of MAP1S and autophagy markers are coincident or have any cause-effect relationship, we treated two pancreatic cancer cell lines with TGFβ to test its impact on the levels of MAP1S and autophagy markers. Real-time PCR analyses revealed that TGFβ had no significant impact on the expression of MAP1S gene in pancreatic cancer cell line PANC-1 and Capan-2 (Fig 4A). Immunoblotting analyses indicated that levels of MAP1S protein were enhanced upon TGFβ treatment (Fig 4B and 4C). Levels of LC3-II were elevated upon TGFβ treatment (Fig 4B and 4D), suggesting two different types of impacts: an enhancement of biogenesis of autophagosomes or an impairment of degradation of autophagosomes. If TGFβ enhances the biogenesis of autophagosomes, levels of LC3-II will be higher than the untreated control in the presence of lysosomal inhibitor Bafilomycin A1. If TGFβ impairs the degradation of autophagosomes, levels of LC3-II will be the same as or lower than those in the controls in the presence of Bafilomycin A1. Levels of LC3-II were elevated in the presence of lysosomal inhibitor Bafilomycin A1 (Fig 4B and 4D), suggesting a general enhancement of autophagy flux by TGFβ. The enhancement of autophagy flux by TGFβ was confirmed by an increase in the number of GFP-LC3 punctate foci upon TGFβ treatment (Fig 4E and 4F). Therefore, TGFβ enhances levels of MAP1S protein and autophagy flux.

### The enhancement of autophagy flux by TGFβ depends on the levels of MAP1S protein

**Fig 5:**
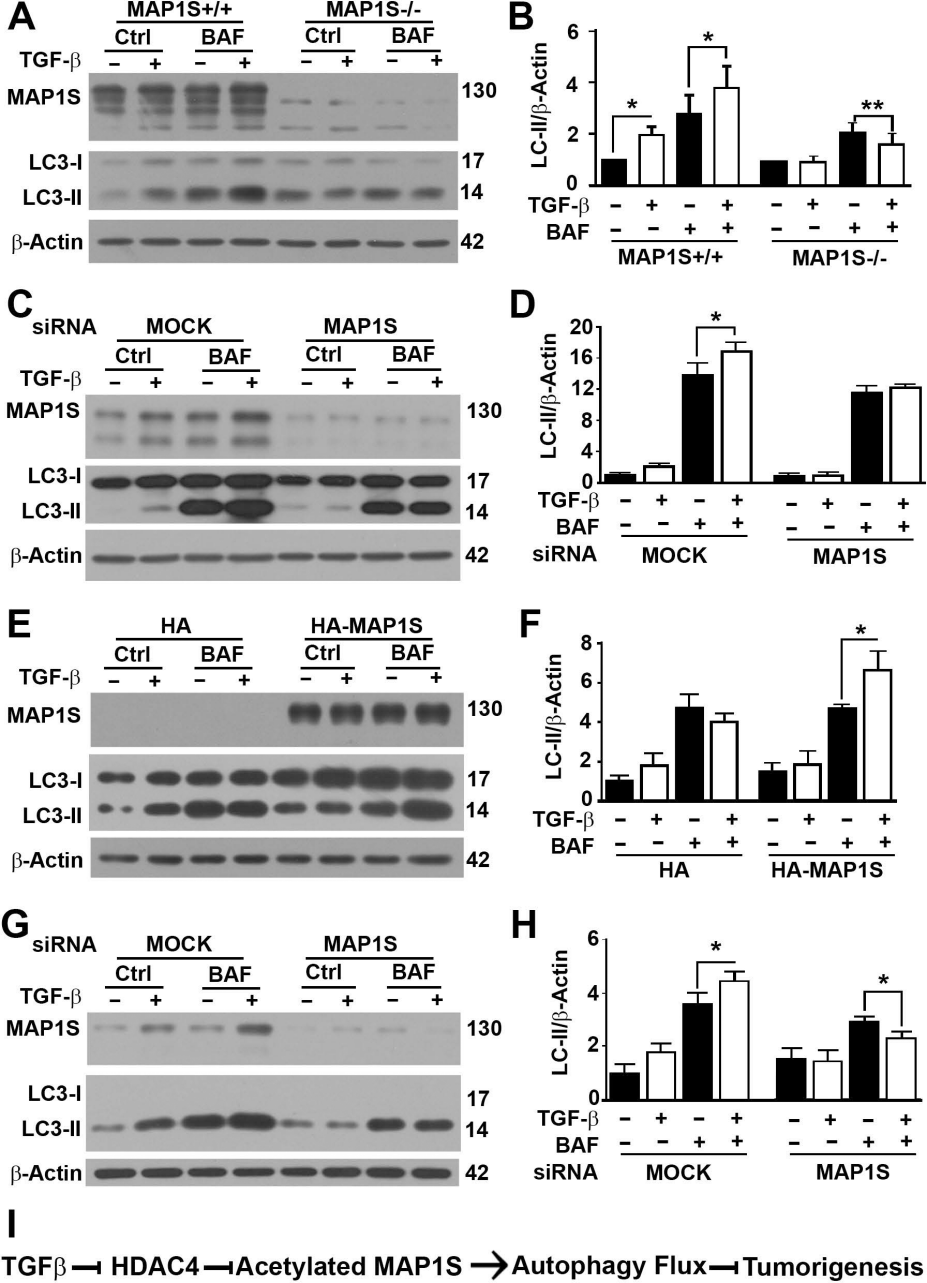
The enhancement of autophagy flux by TGFβ depends on the protein levels of MAP1S. A,B) Immunoblotting analyses (A) and plots (B) of the impact of TGFβ on autophagy flux marker LC3-II in MEF cells developed from wild-type (MAP1S+/+) or MAP1S knockout mice (MAP1S-/-) in the absence (Ctrl) or presence of Bafilomycin A1 (BAF). C,D) Immunoblotting analyses (C) and plots (D) of the impact of TGFβ on autophagy flux marker LC3-II in MEF cells developed from wild type mice (MAP1S+/+) treated with mixture of random sequence siRNAs (MOCK) or MAP1S-specific siRNA (MAP1S) in the absence or presence of BAF. E,F) Immunoblotting analyses (E) and plots (F) of the impact of TGFβ on autophagy flux marker LC3-II in MEF cells developed from knockout mice (MAP1S-/-) transiently transfected with empty vector (HA) or plasmid carrying HA-MAP1S in the absence or presence of BAF. G,H) Immunoblotting analyses (G) and plots (H) of the impact of TGFβ on autophagy flux marker LC3-II in Capan-2 cells treated with mixture of random sequence siRNAs (MOCK) or MAP1S-specific siRNA (MAP1S) in the absence or presence of BAF. The same amounts of total proteins were loaded and β-Actin served as another control. The relative intensities of LC3-II in untreated wild type cells were set as 1. Data were the means ± S.D. of at least three repeats and the significance of differences was tested by Student’s t test. *, P≤0.05; **, P≤0.01; and unlabeled, not significant. I) A diagram showing the mechanism by which TGFβ suppresses tumorigenesis through MAP1S-mediated autophagy flux.

To analyze whether the enhancement of autophagy flux by TGFβ depended on MAP1S, we tested the impact of TGFβ on autophagy flux in MEF cells developed from wild-type and MAP1S-deficient mice. The enhancement of autophagy flux by TGFβ was only observed in wild-type MEF cells but not in MAP1S-deficient MEF cells (Fig 5A and 5B). When the MAP1S expression in wild-type MEF cells was suppressed with MAP1S-specific siRNA, the impact of TGFβ on autophagy flux disappeared (Fig 5C and 5D). When the MAP1S expression in MAP1S-deficient MEF cells was restored by overexpressing MAP1S, the response to TGFβ was rescued although the positive impact of MAP1S alone on autophagy flux was not recovered (Fig 5E and 5F). When the MAP1S expression in Capan-2 cells was suppressed with human MAP1S-specific siRNA, the enhancive impact of TGFβ on autophagy flux was reversed to become suppressive (Fig 5G and 5H). Therefore, TGFβ activates autophagy flux through MAP1S protein.

## Discussion

TGFβ was originally identified and named so based on its ability to induce malignant behavior of normal fibroblasts [34]. The functions of TGF-β signaling pathway has expanded to regulate cell growth, differentiation, apoptosis, cell motility, extracellular matrix production, angiogenesis and cellular immune response [35]. TGF-β acts as a tumor suppressor at the early stage but promotes metastasis at late stage of the development of pancreatic ductal adenocarcinomas [36]. Although the TGFβ signaling pathway emerges as a main regulator of pancreatic tumorigenesis [23], the prognostic significance of TGF-β ligand itself in pancreatic ductal adenocarcinomas was conflictingly reported [36]. Some patients with resectable pancreatic ductal adenocarcinoma have high levels of TGFβ and survive for long time [27]. The relationship between TGFβ and survival remains unknown.

Autophagy was proposed to act like a double-edged sword and play either a promoting or a suppressive role in tumorigenesis [37]. Recently, the tumor promoting function of autophagy was obviously emphasized since several clinical trials utilizing autophagy and lysosome inhibitor hydroxychloroquine for therapy of different types of cancers including metastatic pancreatic adenocarcinoma were conducted [38–41]. However, a suppressive impact of autophagy on pancreatic ductal adenocarcinoma was also reported [42]. Therefore, the relationship between autophagy and tumorigenesis is far from being clarified.

The tumor suppressive function of autophagy regulator MAP1S and its interactive protein LRPPRC has been documented in multiple types of cancers including human ovarian cancer [19], prostate cancer [21, 22], gastric cancer [43] and carcinogen-induced mouse hepatocellular carcinomas [6, 20]. Here, we further show that MAP1S-mediated autophagy is activated in pancreatic cancer patients. We reason that in response to metabolic stress triggered by development of pancreatic cancer, MAP1S-mediated autophagy flux is activated to suppress genome instability and tumorigenesis. If the autophagy flux is blocked, more malignant tumor will develop in a similar way as we reported in the MAP1S knockout mice [6, 20]. TGF-β was reported to be activator of autophagy flux and suppress the development of human hepatocellular carcinoma [28]. Similarly, we show that levels of TGF-β are enhanced in the tumor tissues from pancreatic cancer patients and may suppress the development of pancreatic cancer.

It was reported that the mRNA levels of Beclin 1, ATG5 and ATG7 were elevated several folds but their protein levels remained unchanged when HuH7 cells were treated with TGFβ [28]. Although the possibility for TGFβ to enhance autophagy flux through Beclin 1, ATG5 and ATG7 cannot be excluded, we believe that it is not convincing to assert so if only the mRNA levels of autophagy regulatory genes are elevated since autophagy is a process mediated by a machinery made of proteins. In addition to affecting autophagy through Beclin 1, ATG5 and ATG7, TGFβ may more likely enhance autophagy flux through MAP1S because the protein levels of MAP1S were dramatically elevated. TGFβ was reported to inhibit the activity of Histone deacetylase 4 (HDAC4) [44, 45]. HDAC4 interacts with MAP1S [46, 47]. HDAC4 regulates the acetylation and stability of MAP1S to impact autophagy flux [47]. It is clear that TGFβ increase the levels of endogenous MAP1S to enhance autophagy flux (Fig 5I). However, overexpression of MAP1S in MAP1S^-/-^ MEF cells did not rescue the positive effect of MAP1S on autophagy flux (Fig 5E and 5F). We reason that it is the acetylated MAP1S that activates autophagy. Due to a balance between acetylation and deacetylation of MAP1S, levels of acetylated MAP1S and autophagy flux remain unaltered even when MAP1S is overexpressed. Further treatment with TGFβ causes HDAC4 inhibition that may increase the levels of acetylated MAP1S to enhance autophagy flux. Future work will be concentrated on investigating the mechanism by which TGFβ enhances the levels of MAP1S and its mediated autophagy flux in depth.
